# An Unusual Congenital *In Situ* Malrotation of the Liver

**DOI:** 10.1155/2013/493713

**Published:** 2013-12-03

**Authors:** Hua Zhong

**Affiliations:** Department of Pathology and Laboratory Medicine, Rutgers Robert Wood Johnson Medical School, Rutgers Cancer Institute of New Jersey, 195 Little Albany Street, Room 2045, New Brunswick, NJ 08903, USA

## Abstract

Congenital anomaly of the liver is an uncommon and usually incidental finding. This report describes a case of *in situ* liver malrotation that has never been reported in the literature. Published literature relevant to the finding are briefly discussed.

## 1. Introduction

Congenital deviations from the usual anatomy in the liver are uncommon. Some of such anomalies may be associated with severe genetic alterations or complex developmental defects and may eventually jeopardize liver function. Anatomical anomalies of the liver, for example, situs anomalies, or abnormal lobation including accessary lobe, in particular those causing little functional problem, can be detected in pediatric and adult patients during imaging exams [1], surgical procedures [2], or autopsies [3]. Recognizing various anatomical abnormalities of the liver will benefit clinical practice, especially valuable for radiologists and surgeons. Herein, a case of unusual anatomic anomaly of the liver is reported in an adult as an incidental finding during autopsy.

## 2. Case Report

**Figure 1:**
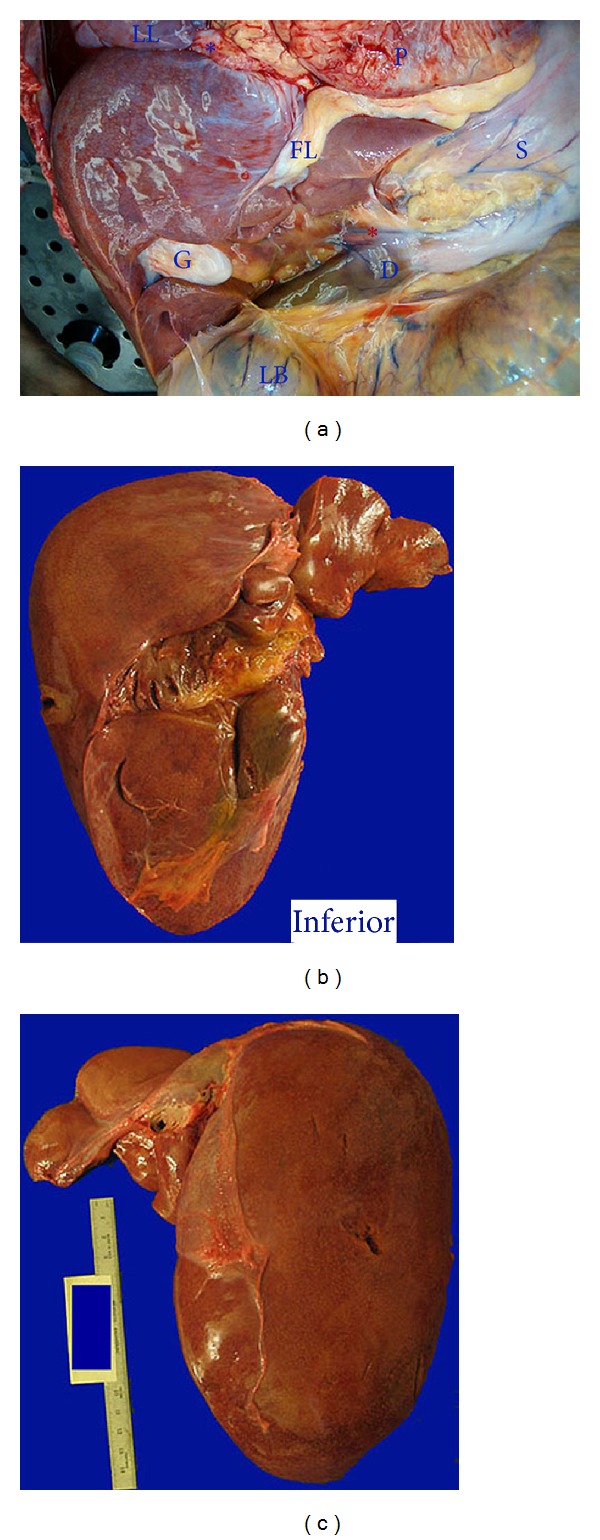
(a) *In situ* view of the liver in relation to adjacent organs. The chest and abdominal wall were opened, and the diaphragm was removed. The remnant of the diaphragm is marked with a blue [∗]. A red [∗] denotes hepatoduodenal ligament; LL denotes left lung; FL, falciform ligament; P, pericardium; S, stomach; D, duodenum; G, gallbladder; and LB, large bowel. (b) Anterior view of the liver. (c) Posterior view of the liver.

A 39-year-old African American female with no abdominal surgical history passed away due to catastrophic antiphospholipid syndrome secondary to active systemic lupus erythematosus with systemic thrombosis involving multiple organs including the lungs. The cause of death was supported by clinical presentation and laboratory studies which was confirmed by authorized autopsy. In addition, an unusual anatomical anomaly of the liver was demonstrated. The liver appeared to be malrotated *in situ*, with the inferior aspect and the gallbladder facing anteriorly (Figure 1(a)). The right lobe was unusually prominent, with the largest dimension of 23 cm from superior to inferior. The left lobe appeared to be atrophic (Figures 1(b) and 1(c)). The liver weighed 1,300 grams. The falciform ligament and the round ligament were thickened, whereas the bilateral triangular ligaments, the hepatogastric ligament, and the coronary ligament had become atrophic. The hepatogastric ligament was shifted to the left edge of the left lobe. The ligamentum venosum was completely obliterated. The hepatoduodenal ligament was identified and contained the common bile duct and the hepatic vessels. However, the ligament appeared to abnormally be away from the gallbladder neck region. Consecutive sectioning revealed uniform red-brown parenchyma with no gross fibrosis, nodules, tumor masses, or intravascular thrombi. The anteriorly located gallbladder was flattened and small, with a whitish glistening surface and thickened wall (Figure 1(a)). The gallbladder contained little viscous green bile. Other organs seemed to bear the correct anatomical positions. Microscopically, the liver parenchyma showed diffuse sinusoidal dilatation and congestion, mild zone three collapse, and mild lymphocytic portal tract infiltrates. Microscopic thrombi were not identified in multiple random sections. No evidence of nodular regenerative hyperplasia was demonstrated. The microscopic findings were consistent with the patient's systemic pathology and did not seem to be specific for the anatomic anomaly of the liver.

## 3. Discussion

Anatomical anomaly in the adult liver is a very rare occurrence. The majority of them usually involve a part of the liver, a lobe, or segments that present with agenesis or fusion [2]. The left lobe atrophy, as demonstrated in the current case, is a relatively common structural abnormality. In pediatric population, anatomical anomalies of the liver are usually found as one component of a complex congenital defect that often involves adjacent organs, for example, different types of situs anomaly [1]. Liver malrotation is extremely rare. It was once reported in an adult patient with congenital diaphragmatic hernia, coupling the positional change (displacement) of the liver [4]. To my knowledge, the unique *in situ* malrotation in the current case has never been reported in the literature. Unfortunately, more extensive dissection and sampling were not performed during the autopsy process; otherwise, this report could be more informative.
